# Protocol for establishing a chemoresistant orthotopic triple-negative breast cancer patient-derived xenograft model for preclinical drug testing

**DOI:** 10.1016/j.xpro.2026.104394

**Published:** 2026-03-02

**Authors:** David Tébar-García, Rosa Barbella-Aponte, Esther Sánchez-López, Maria G. Picazo-Martínez, Mónica Gómez-Juárez, Ángela Díaz-Piqueras, Alejandro Pinedo-Serrano, Paula Sánchez-Olivares, Raquel López-Rosa, Pablo Cristobal-Cueto, Verónica Rodilla, Eva M. Galán-Moya

**Affiliations:** 1 Cancer Pathophysiology and Therapy Lab Institute of Biomedicine of the University of Castilla-La Mancha (IB-UCLM), Albacete, Spain; 2 Anatomic Pathology Service, Albacete University Hospital, Albacete, Spain; 3 Surgical Unit, Albacete University Hospital, Albacete, Spain; 4 Experimental Research Unit of General University Hospital of Albacete, Spain Albacete, Spain; 5 Biobank, Albacete University Hospital, Albacete, Spain; 6 Cancer Heterogeneity and Hierarchies Group, Josep Carreras Leukaemia Research Institute (IJC), Badalona, Spain

**Keywords:** Cancer, Model Organisms, Biotechnology and bioengineering

## Abstract

Here, we present a protocol for establishing patient-derived xenograft (PDX) mouse models of chemoresistant triple-negative breast cancer (TNBC). We describe steps for tumor collection, surgical implantation, and serial expansion. We then detail procedures for histopathological validation and *in vivo* therapeutic assessment to confirm the maintenance of chemoresistance across passages. These models provide a clinically relevant platform to investigate mechanisms of treatment resistance, evaluate novel therapeutic strategies in preclinical settings, and predict therapeutic responses in refractory TNBC.

## Highlights

• Establishment of chemoresistant TNBC patient-derived xenograft (PDX) models
• Orthotopic implantation into the mammary fat pad of immunodeficient NSG mice
• Serial passaging to preserve tumor identity and treatment resistance
• Preclinical drug evaluation maintaining tumor biology and resistance phenotype

## Abstract (editor highlights)

Publisher’s note: Undertaking any experimental protocol requires adherence to local institutional guidelines for laboratory safety and ethics.

## Graphical abstract

**Figure:**
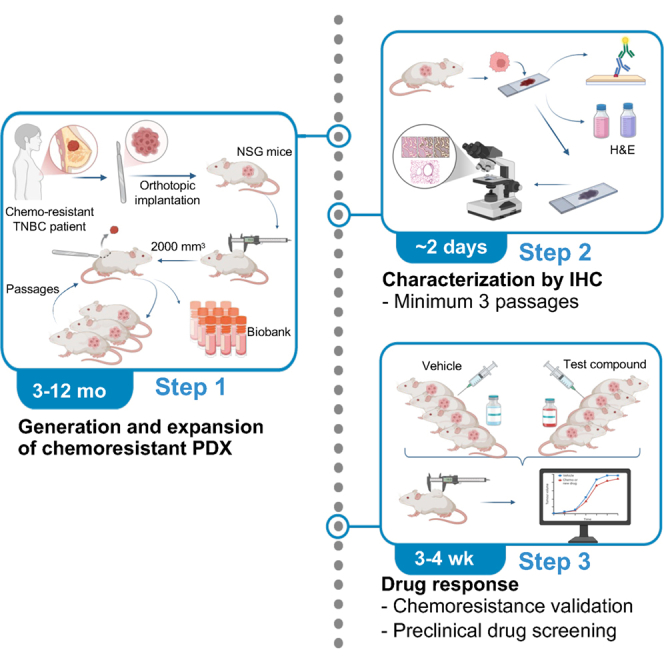

## Teaser

Here, we present a protocol for establishing patient-derived xenograft (PDX) mouse models of chemoresistant triple-negative breast cancer (TNBC). We describe steps for tumor collection, surgical implantation, and serial expansion. We then detail procedures for histopathological validation and *in vivo* therapeutic assessment to confirm the maintenance of chemoresistance across passages. These models provide a clinically relevant platform for investigating mechanisms of treatment resistance, preclinical testing of novel therapeutic strategies, and predicting therapeutic responses in refractory TNBC.

## Before you begin

### Innovation

Triple-negative breast cancers (TNBC) are defined by the absence of estrogen receptor (ER) and progesterone receptor (PR), which mediate hormone-driven tumor growth, and by the lack of epidermal growth factor receptor 2 (HER2) overexpression, a key driver targeted by anti-HER2 therapies. Consequently, TNBC are insensitive to endocrine and HER2-targeted treatments, display an aggressive tumor behavior and rapid proliferation, and rely primarily on chemotherapy as the main systemic treatment option.

This protocol details the establishment of patient-derived xenograft (PDX) models from chemoresistant TNBC tumors using orthotopic implantation in immunodeficient NSG mice. The resulting PDXs preserve the histopathological features, molecular markers, and lack of response to neoadjuvant chemotherapy observed in the original patient tumors, providing a clinically relevant *in vivo* platform for studying refractory disease.

By focusing specifically on tumors with confirmed clinical chemoresistance, and by incorporating serial passaging with phenotypic validation, this protocol ensures the maintenance of the resistant phenotype across generations. This represents a critical improvement over conventional PDX workflows, where therapeutic resistance is often not well defined or is gradually lost.

Therefore, this protocol fills an important gap in the field by enabling the systematic development of clinically representative TNBC resistance models. It provides a robust translational platform for investigating chemoresistance pathways or for evaluating new therapeutic strategies in patients with refractory TNBC, supporting both mechanistic research and preclinical drug development.

### Institutional permissions

All clinical sample collection must be completed after obtaining approval from the corresponding ethics committee and informed consent from all participants. Human samples used in this study were provided by the Albacete Hospital Biobank, collected in accordance with institutional guidelines and with the approval of the Ethics Committee for Investigation with Medicinal Products of General University Hospital of Albacete (approval number 09/2022-087).

All animal procedures must receive prior approval from the Ethical Committee for the Use of Experimental Animals. Animal experiments in this study were performed following the European Directive 2010/63/UE and Spanish legislation (Real Decreto 53/2013) for the protection of animals used for scientific purposes. Animal studies were approved by the Animal Experimentation Ethics Committee of Castilla-La Mancha University (PR-2020-07-10) and were carried out at the Experimental Research Unit of General University Hospital of Albacete.

### Animal preparation

1. Purchase three founder pairs of NSG mice (3 males and 3 females, 35-41 days old) from a commercial breeder to stablish an NSG breeder colony.
2. Identify and house males and female separately and collect female NSG mice when they reach 5–6 weeks of age.

***Note:*** NSG mice are widely validated for breast cancer PDX generation and support robust tumor engraftment and serial passaging; therefore, this strain was used throughout the protocol to ensure reproducibility.

***Optional:*** Alternatively, purchase 5-6-week-old female NSG mice directly from an accredited commercial breeder.

3. House animals under stardard husbandry conditions:
  a. Group-house mice (5–6 per cage) in climate-controlled, individually ventilated cages containing aspen-chip bedding.
  b. Maintain a 12:12 h light–dark cycle.
  c. Maintain ambient temperature at 22 ± 2°C and relative humidity of 30%–70%.
  d. Provide *ad libitum* access to irradiation-sterilized food and water.
4. Maintain mice under Specific Pathogen-Free (SPF) conditions within the animal facility.
5. Prepare all solutions listed in the materials and equipment section before starting each.

### TNBC tissue preparation

**Timing: 4–24 h**

6. Collect and document the patient tumor sample:
  a. Collect the sample directly from the responsible clinician.
  b. Confirm that the tissue is diagnostic overage.
  c. Record the collection date, clinician’s name, patient identifier, and sample type in the Sample Collection Log.
7. Remove large blood vessels, necrotic areas, and excess stroma under sterile conditions.

***Note:*** Necrotic tissue in breast tumors typically appears white, whereas viable tissue is beige to yellow and firmer in texture.

**CRITICAL:** Thoroughly remove all necrotic regions to maximize engraftment success and ensure viable tumor propagation.

8. Wash the tumor twice in PBS supplemented with penicillin/streptomycin (P/S), ciprofloxacin and amphotericin B (materials and equipment Section) for 5–10 min.
9. Incubate the sample for at least 2 h in DMEM (phenol-red free) containing P/S, ciprofloxacin, and amphotericin B to reduce microbial contamination.

***Optional:*** The tumor may be stored 16 h in this medium at 4°C. If thawing cryopreserved material, thaw directly in FBS with antibiotics and incubate 16–24 h at 4°C.

10. In a laminar-flow hood, cut the tumor into ∼3 × 3 mm fragments using a sterile scalpel.

***Optional:*** At this stage, tumor fragments can be cryopreserved at −80°C or in liquid nitrogen using the freezing medium described in the Materials and equipment setup Section.

***Note:*** The use of freshly collected samples generally improves efficiency of engraftment.

## Key resources table

**Table:** 

REAGENT or RESOURCE	SOURCE	IDENTIFIER
**Antibodies**		

Progesterone Receptor, Clone PgR 1294, FLEX Ready-to-Use (RTU). Monoclonal Mouse Anti-Human.	Agilent Dako	#Cat. GA090
Estrogen Receptor α, Clone EP1, FLEX RTU. Monoclonal Rabbit Anti-Human.	Agilent Dako	#Cat. GA084
Ki-67 Antigen, Clone MIB-1, FLEX RTU. Monoclonal Mouse Anti-Human.	Agilent Dako	#Cat. GA626

**Biological samples**		

Patient tumor samples of Breast cancer: Female adults (>18) and refractory to neoadjuvant	University Hospital of Albacete (CHUA)	–

**Chemicals, peptides, and recombinant proteins**		

PBS	Corning	#Cat. 21-031-CV
DMEM, phenol red free	Corning	#Cat. 17-205-CV
FBS heat-inactivate	Corning	#Cat. 35-079-CV
Penicillin-Streptomycin (P/S)	Corning	#Cat. 30-002-CI
Amphotericin B	Gibco	#Cat. 15290026
Ciprofloxacin	Normon	C.N 603181 E.C.
Dimethyl Sulfoxide(DMSO)	Fisher BioReagents	#Cat. BP231-1
Amoxicilin	Norton	#Cat. 823187.4
Sevoflurane	Baxter	#Cat. 669629.3
Bupivacaine	Physan	#Cat. 607371
Nab-paclitaxel (Abraxane)	Bristol Myers Squibb	EU/1/07/428/001
Water for injectable preparations	Fresenius Kabi	C.N. 796748
Surgipath Paraplast Bulk	Leica	#Cat. 39602012
Hematoxylin	Agilent Dako	#Cat. GC808
Eosin	Agilent Dako	#Cat. CS701
Clearify Clearing Agent	Agilent Dako	#Cat. GC810
EnVision FLEX Target Retrieval Solution High pH (50x)	Agilent Dako	#Cat. GV804
Wash Buffer (20x)	Agilent Dako	#Cat. GC807
EnVision FLEX Peroxidase Blocking Reagent High pH	Agilent Dako	#Cat. GV800
EnVision FLEX+ Mouse LINKER	Agilent Dako	#Cat. GV821
HercepTest for Automated Link Platforms, Kit system, 50 tests. Includes: - HercepTest Rabbit Anti-Human HER2 primary antibody - HercepTest Visualization Reagent	Agilent Dako	#Cat. SK001
EnVision FLEX DAB+ Substrate Chromogen System	Agilent Dako	#Cat. GV825

**Experimental models: Organisms/strains**		

Mouse: NSG (NOD.Cg-Prkdcscid Il2rgtm1WjI/SzJ) female mice NOD/ShiLtJ background; 5–6 weeks	Charles River	Cat#614

**Other**		

Triangular silk 5 0s 1/2 21 mm	Silkam	#Cat. C0764876
Formaldehyde 3.7%–4.0% w/v buffered to pH=7 and stabilized with methanol	Panreac	#Cat. 252931
Microscope slides 76 X 26 MM	Knittel Glass	N/A
Flex IHC Microscope Slides	Dako	#Cat. K8020
MX35 Ultra Microtome Blade	Eprendria	#Cat. 3053835
Syringe 1 mL	BD Plastipak	#Cat. 303172
Sterican Safety Needle 27G x ½”	Braun	#Cat. 4670005S-01
Magnus Lean High Throughput Tissue Processor	Milestone	N/A
Leica HistoCore Arcadia H	Leica	N/A
Microm HM 355S	Thermo Scientific	N/A
Dako CoverStainer	Dako	RRID: SCR_019496
Dako Omnis	Dako	RRID: SCR_019495
Dako AutoStainerLink 48	Dako	N/A
Zeiss PrimorStar 3 – Axioncam 208 Color P90-C ½” 0.5x	Zeiss	N/A

**Software and algorithms**		

BioRender	BioRender	https://BioRender.com/kgdw51z RRID:SCR_018361
Prism 8	GraphPad	https://www.graphpad.com/scientific-software/prism/

## Materials and equipment

***Note:*** All antibodies listed in the key resources table were used in a ready-to-use (RTU) format according to the manufacturers’ instructions and did not require dilution.

### Freezing medium

Tumor freezing medium preserves cell viability and tissue integrity during long-term storage at −80°C or in liquid nitrogen. It prevents crystal formation and enables recovery of viable tissue upon thawing.

**Table:** 

Reagent	Final concentration	Amount
FBS	95% v/v	950 μL
Dimethyl Sulfoxide (DMSO)	5% v/v	50 μL

Mix gently and cool before use.

**CRITICAL:** The preparation is exothermic; allow the solution to equilibrate to 20°C–25°C before contact with tissue.

Store at 4°C for up to 1 month. Prepare beforehand since it is an exothermic reaction and must be cooled beforehand. Prior to freezing, it should be tempered to 37°C before contacting the tumor pieces.

### DMEM or PBS with antibiotics

This medium is prepared to preserve extracted tumors and prevent contamination before implantation in PDX models. It contains antibiotics such as P/S, ciprofloxacin, and amphotericin to eliminate bacteria and fungi, ensuring tissue viability and reducing the risk of infection. This is the same priority that must be prepared with PBS, since the concentrations are the same.

**Table:** 

Reagent	Final concentration	Amount
DMEM F-12 without phenol red or PBS	87.5% v/v	43.75 mL
P/S	5% v/v	2.5 mL
Ciprofloxacin	5% v/v	2.5 mL
Amphotericin	2.5% v/v	1.25 mL

***Note:*** Mix under sterile conditions and store at 4°C for up to 2 weeks.

## Step-by-step method details

### Generation and expansion of resistant PDXs

**Timing: 3–12 months**

This section describes the generation and serial propagation of PDXs from TNBC samples obtained from patients who relapsed following neoadjuvant chemotherapy.

**Figure 1:**
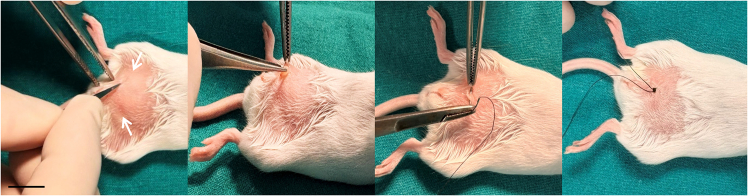
Subcutaneous implantation of a chemoresistant TNBC PDX into the mammary fat pad of NSG mice Representative images illustrating the surgical procedure for orthotopic PDX implantation. From left to right: shaving of the lower mammary line and skin incision, insertion of the PDX tumor fragment, closure of the incision prior to suturing, and final wound closure with visible sutures. Scale bar: 10 mm.
4. Administer amoxicillin short-term in drinking water (0.33 mg/mL for 7 days post-surgery).

***Note:*** Amoxicillin treatment is used as a prophylactic measure to reduce the risk of infection in immunocompromised mice. This exposure can induce transient microbiota alterations that typically recover within ∼3 weeks and can be omitted or adjusted according to institutional guidelines.

1. Prepare the surgical area in a biosafety cabinet or designated sterile operating space.
  a. Clean all surfaces and instruments with appropriate disinfectants.
  b. Ensure all surgical tools (needle holders, scissors, forceps) are sterilized and autoclaved.
  c. Pre-warm the thermal pad to 39°C before surgery.
2. Verify that anesthetic and postoperative care materials (sevoflurane, bupivacaine, amoxicillin) are ready and sterile.
3. Proceed to the surgical procedure in mice (Figure 1 and Methods Video S1).
  a. Induce anesthesia in a chamber containing a mixture of oxygen and 5% sevoflurane at a flow rate of 1 mL/s for ∼3 min. Confirm anesthesia by loss of righting reflex.
  b. Transfer the anesthetized mouse to the thermal pad in a supine position. Maintain anesthesia with 2%–3% sevoflurane via a face mask.
  c. Shave and disinfect the ventral area of the mice.
  d. Administer bupivacaine (2 mg/kg) subcutaneously at the incision site using a 27 G needle.
  e. Make a minimal incision (∼2–3 mm) adjacent to the inguinal mammary gland (4^th^ or 5^th^ mammary gland pair) to expose the mammary fat pad.
    ***Note:*** The inguinal fat pad extends between the 4th and 5th mammary glands and provides a suitable site for orthotopic engraftment. Thoracic mammary glands are not recommended, as the small size of the thoracic fat pad and its proximity to the ribs may restrict tumor expansion. Bilateral implantation (left and right glands) may be used for tumor expansion or passaging.^1^
  f. Using sterile forceps, insert a tumor fragment (∼3–4 mm^3^) on top of the exposed mammary gland.
    ***Optional:*** Single-cell suspension–based implantation can be used as an alternative approach; however, due to the dense extracellular matrix of TNBC tumors, this may require extensive enzymatic dissociation and can reduce tumor heterogeneity and resistance-associated features.
  g. Close the incision with non-absorbable silk sutures (5/0).
    **CRITICAL:** Avoid pressing or damaging the tumor fragment during placement. Ensure the incision is fully closed to prevent dehydration or infection.
    Methods Video S1. Orthotopic implantation of a TNBC tumor fragment into the mammary fat pad of an NSG mouse (Steps 3e-g) The video demonstrates surgical exposure of the mammary fat pad, placement of an intact tumor fragment onto the gland, and closure of the incision, highlighting key steps to avoid tissue damage and ensure successful engraftment.

5. Monitor animals daily for general health, surgical recovery, and signs of infection (problem 1).
6. Measure tumor growth weekly using calipers and calculate with tumor volume using the formula: $\left(\frac{Length\;x\;{\left(Width\right)}^{2}}{2}\right)$.
7. Euthanize animals when tumor volume reaches 1,500–2,000 mm^3^.
8. Aseptically resect the tumor, cut into fragments, and use for serial transplantation in new mice.

***Note:*** The time from implantation to tumor establishment varies depending on tumor aggressiveness and stromal composition (typically 3–12 months) (Table 1) (problem 2).

9. At each passage:
  a. Divide the tumor tissue into fragments and implant portions into 3–5 NSG recipient mice per passage, depending on tissue availability.
    ***Note:*** Engraftment success is defined by the formation of a measurable tumor at the implantation site.
  b. Fix one portion in 4% formaldehyde for 24 h at 20°C–25°C for histological analysis.
  c. Freeze one portion for molecular assays (e.g., Western blot, qPCR, RNA-seq).
  d. Cryopreserve 3×3 mm fragments in freezing medium for biobanking (see materials and equipment).
    **CRITICAL:** Define the PDX as established after three successful serial passages showing consistent tumor engraftment and growth (P3). At this point, the PDX model is ready to proceed to preclinical studies^2^ (problem 3).
10. To biobank tumor fragments:
  a. Prepare freezing medium 1–2 h in advance and chill at 4°C.
  b. Transfer 3 × 3 mm tumor fragments into cryovials containing 1 mL of pre-cooled freezing medium.
  c. Place cryovials in a controlled-rate freezing container and store at −80°C overnight.
  d. For long-term storage, transfer to liquid nitrogen.

**CRITICAL:** Always label cryovials with passage number, patient ID, and date to maintain traceability.

**Table 1:** Average time required for PDX tumors to reach a volume of 2,000 mm^3^ across passages 1, 2, and 3

Passage	Average growth time
P1	141 days
P2	70 ± 24 days
P3	65 ± 3 days

The table summarizes the mean number of days required for tumors from successive PDX passages to reach endpoint volume, illustrating progressive adaptation to the murine microenvironment.

### Immunohistochemical evaluation

**Timing: 24 h fixation and 7–8 h staining protocol**

This section describes the histological processing and immunohistochemical (IHC) characterization of PDX tumors to verify their correspondence with the original patient samples.

11. Tissue fixation and processing:
  a. Fix tumor fragments in 4% formaldehyde for 24 h at 20°C–25°C.
  b. Wash the fixed tissue twice with 1× PBS, then transfer to 70% ethanol.
  c. Load the specimen into a Magnus Lean High-Throughput Tissue Processor for dehydration, clearing, and paraffin infiltration:
    i. Sequential immersion in 70%, 80%, 96%, and 100% ethanol (1–2 min each).
    ii. Two incubations in xylene (90 s each).
    iii. Two incubations in molten paraffin (2 min each).
  d. Embed the tissue in paraffin (paraplast) using a Leica HistoCore Arcadia H embedding station.
  e. Pour molten paraffin into labeled cassettes and orient tissue centrally.
  f. Allow blocks to solidify at 20°C–25°C.
  g. Cut 3 μm sections using a Microm HM 355S microtome and mount on FLEX IHC microscope slides.

**CRITICAL:** Ensure all tissues are completely dehydrated before embedding to prevent tissue detachment during sectioning or staining.

**Figure 2:**
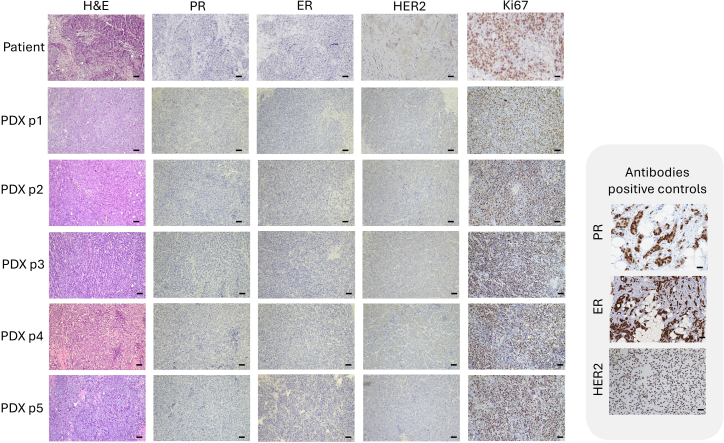
Immunohistochemical characterization of the original chemoresistant TNBC tumor and derived PDXs Representative immunohistochemical staining for hematoxylin and eosin (H&E), progesterone receptor (PR), estrogen receptor (ER), HER2, and Ki-67 in the original patient tumor and corresponding PDX tumors from passages 1 to 5 (P1–P5). Representative positive controls are included for PR, ER, and HER2. Scale bar: 150 μm.

12. Hematoxylin and eosin (H&E) staining:
  a. Dewax slides in xylene (2 × 3 min).
  b. Rehydrate sequentially through graded ethanol (100%, 96%, 70%) to distilled water.
  c. Stain with hematoxylin for 4.5 min, rinse in tap water (30 s), and blue in running water (2 min).
  d. Counterstain with eosin for 5 min, dehydrate through 70%, 96% and 100% ethanol, and clear in Histoclear (2 × 2 min).
  e. Mount slides with coverslips automatically using the Dako CoverStainer system.
13. Immunohistochemical staining protocols:
  a. Perform IHC on 3 μm sections using the Dako Omnis automated staining platform.
  b. Primary antibodies include ERα (EP1 clone), PR (1294 clone), Ki-67 (MIB-1 clone), and HER2 (HercepTest™ pharmDx kit).
    ***Note:*** All antibodies were used in a RTU format according to the manufacturer’s instructions.
  c. Use EnVision™ FLEX reagents according to manufacturer’s instructions.
    ***Note:*** For reproducibility, all IHC runs should include positive and negative controls processed in parallel. Positive control tissues (e.g., ER-positive, PR-positive, or HER2-positive breast carcinoma) known to express the target antigen were used for ER, PR, and HER2 antibodies.
14. ER, PR, and Ki-67 staining (Dako Omnis platform):
  a. Perform two-phase dewaxing using Clearify Clearing Agent in deionized (DI) water at 25°C (10 s short zone, 60 s intermediate zone).
  b. Antigen retrieval: incubate in EnVision™ FLEX TRS (High pH) at 95°C for 30 min; cool with DI water.
  c. Wash slides twice with wash buffer (160 s each).
  d. Incubate with primary antibody (ERα EP1, PR 1294, or Ki-67) for 15 min at 20°C–25°C.
  e. Wash 10× for 2 min each with wash buffer.
  f. Block endogenous peroxidase activity using EnVision™ FLEX Peroxidase Blocking Reagent for 3 min.
  g. For PR, add EnVision™ FLEX+ Mouse LINKER for 10 min before proceeding at 20°C–25°C.
  h. For all antibodies, incubate with EnVision™ FLEX/HRP for 20 min.
  i. Wash slides 20× (2 min each) in wash buffer, followed by DI water rinse (30 s).
  j. Develop chromogen using EnVision™ FLEX Substrate Working Solution for 5 min.
15. HER2 staining (HercepTest™ pharmDx, Dako Autostainer Link 48):
  a. Perform dewaxing and antigen retrieval as above (step 14b-c).
  b. Block endogenous peroxidase activity for 3 min.
  c. Incubate with HercepTest™ Rabbit Anti-Human HER2 primary antibody for 10 min at 20°C–25°C.
  d. Apply HercepTest™ Visualization Reagent for 20 min at 20°C–25°C.
  e. Wash slides as above (20× for 2 min each).
  f. Develop signal using EnVision™ FLEX Substrate Working Solution for 5 min.
16. Counterstain with hematoxylin for 3 min, rinse with wash buffer, and mount coverslips using the Dako Omnis automated staining platform or Dako Autostainer Link 48, as appropriate.
17. Image acquisition and analysis (Figure 2):
  a. Examine stained slides under a Zeiss Primostar 3 microscope equipped with an Axiocam 208 color camera.
  b. Capture representative images at 10× magnification to compare morphology and biomarker expression between patient tumors and corresponding PDX passages.
  c. Confirm that PDX tumors maintain the histopathological and immunohistochemical profiles of the original chemoresistant TNBC samples.

### Evaluation of the response to neoadjuvant therapy: Validation of chemoresistance

**Timing: 3–4 weeks (depending on the treatment regime and therapy used)**

This section describes the procedure for validating chemoresistance in established PDX models.

***Note:*** Proceed to treatment when tumors reach 150–200 mm^3^, as this size ensure reliable and measurable assessment of therapeutic response. Because growth rates and final tumor sizes may differ when both left and right mammary glands are used simultaneously, bilateral implantation is not recommended at this stage.

**Figure 3:**
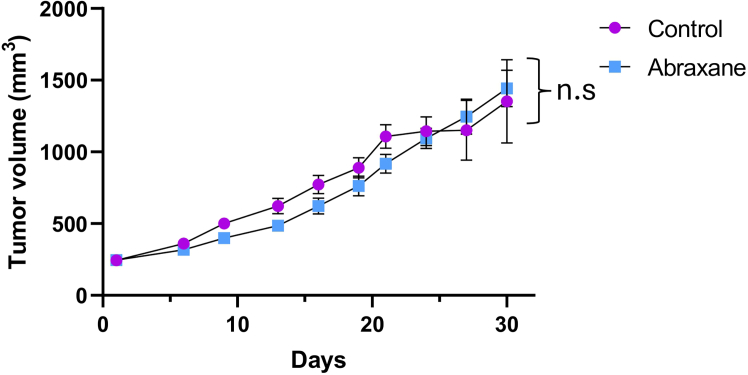
Validation of Nab-paclitaxel resistance in established TNBC PDX models Graph showing mean tumor volume (± standard error of the mean, SEM) in PDX-bearing NSG mice treated with nab-paclitaxel compared with untreated controls. Graph created using Prism 8. Statistical analysis was performed using one-way ANOVA. n.s = non-significant (*p* > 0.05).

***Note:*** Chemoresistant TNBC PDX models show minimal or no tumor regression in response to the specific neoadjuvant chemotherapy received by the patient, maintaining growth kinetics comparable to untreated controls.

18. Preparation of treatment solutions:
  a. Calculate the dose for each animal based on individual body weight (mg/kg or μL/g).
    **CRITICAL:** Convert the human dose to the mouse dose using the FDA-recommended conversion factor: (Mouse (mg/kg) = Human dose (mg/kg) × 12.3)^3^.
  b. Prepare the drug solution by dissolving the compound following the manufacturer’s preparation instructions specific to the compound being tested or an established published protocol, as appropriate.
    ***Note:*** For nab-paclitaxel (albumin-bound paclitaxel), prepare a sterile suspension according to the manufacturer’s preparation protocol ensure proper solubility and bioavailability. For other compounds, formulation and preparation conditions may vary and should follow the corresponding manufacturer’s protocol or a validated *in vivo* formulation. Prepare individual doses immediately before administration.
    **CRITICAL:** Ensure complete the compound is completely dissolved; filter-sterilize if the final solution if necessary.
  c. Load syringes with the exact calculated volume (maximum intraperitoneal [IP] injection volume: 10 mL/kg).
    **CRITICAL:** Confirm the drug is soluble and stable in the selected vehicle. If IP injection is not feasible, use oral or intravenous (IV) routes following appropriate adaptation of the dosing volume (maximum oral gavage volume: 10 mL/kg; maximum IV injection volume: 5 mL/kg).
19. *In vivo* administration and monitoring:
  a. Treat mice when tumors reach a volume of 150–200 mm^3^.
  b. Administer the compound using the appropriate route (IP, IV, or oral) according to the specific drug being tested (e.g., IP injection with a 27 G needle for nab-paclitaxel).
    ***Note:*** Administer the compound slowly to minimize reflux and tissue injury.
  c. Observe each animal for at least 15 min post-injection for signs of pain, distress, bleeding, or abnormal behavior.
  d. Record body weight and clinical observations throughout the treatment period.
  e. Monitor tumor growth twice per week by caliper measurement and calculate volume as above.
  f. Continue treatment at a frequency equivalent to the clinical regimen (e.g., weekly dosing for nab-paclitaxel), adjusting to match cumulative weekly exposure in humans.
    ***Optional:*** If the drug induces inflammatory or hypersensitivity responses, supplement drinking water with dexamethasone (0.006 mg/mL; equivalent to 1 mg/kg) starting 24 h post-treatment for 24 h.
20. Evaluation of chemoresistance (Figure 3):
  a. Compare tumor growth kinetics between treated and control groups.
  b. Plot mean tumor volume ± SEM over time.
  c. Assess chemoresistance as maintenance of tumor growth despite treatment, consistent with the resistant phenotype of the original patient tumor.
  d. For confirmation, repeat drug administration in subsequent passages (e.g., P3–P5) to verify stability of the resistance phenotype (problem 4).

### Preclinical drug screening

**Timing: 2–4 weeks (depending on the treatment regime and drug used)**

This section describes how to evaluate drug efficacy in the established chemoresistant TNBC PDX models using preclinical compounds or approved therapies distinct from the neoadjuvant regime to which the tumors were originally resistant.

***Note:*** Unilateral PDX implantation in either the right mammary gland or left mammary gland (preferred when IP administration is required) is recommended. Proceed to drug screening when tumors reach a volume of 150–200 mm^3^.

21. Administer experimental compounds using the dosing strategy validated in published *in vivo* studies or manufacturer recommendations (see steps 18–19) (problem 5).
22. Define treatment endpoints based on the specific study objective (e.g., ≥50%, tumor volume reduction, delayed tumor growth, or survival benefit).
23. Euthanize animals at the predetermined endpoint in accordance with institutional and ethical guidelines.
24. Collect tumors and relevant organs for histological, molecular, or pharmacodynamic analyses.
25. Store tumor fragments in freezing medium or fix in formalin as described in the step-by-step method details section (step 9).

**CRITICAL:** Record all treatment parameters (dose, route, schedule, vehicle, formulation) for each mouse to ensure reproducibility and allow cross-study comparisons.

## Expected outcomes

During the establishment of patient-derived xenografts (PDXs) from chemoresistant triple-negative breast cancer (TNBC), tumor fragments are expected to preserve the resistant phenotype observed in the corresponding patient tumors. The resulting PDXs typically maintain the histopathological features and molecular marker profile of the primary samples, exhibiting a triple-negative phenotype (ER-, PR-, HER2-) and comparable proliferative indices as determined by Ki-67 immunostaining. Throughout serial passages, these models usually retain the morphological and molecular fidelity of the original tumors, demonstrating stable growth kinetics and reproducible engraftment rates.

Over successive passages in immunodeficient mice, it is common to observe a moderate reduction in the time required for tumors to reach endpoint volume (approximately 2,000 mm^3^).^2^^,^^3^^,^^4^ This decrease reflects an adaptation of the tumor to the murine microenvironment, rather than a change in intrinsic tumor biology, and does not compromise the resistant phenotype. Once the model is established—typically after three successful passages—it can be used for downstream preclinical applications such as drug efficacy testing, resistance mechanism studies, and validation of therapeutic targets.

When treated with Nab-paclitaxel or other chemotherapeutic agents, the established resistant PDXs are expected to exhibit negligible tumor regression, with growth kinetics similar to untreated controls. This behavior confirms the maintenance of chemoresistance in vivo and validates the model as a reliable system to evaluate therapeutic responses in refractory TNBC.

## Limitations

The generation and maintenance of chemoresistant TNBC PDX models involve several inherent limitations that should be carefully considered when planning experiments. One major constraint is the limited availability of suitable patient samples. Tumors resistant to neoadjuvant chemotherapy are relatively uncommon and often obtained only after extensive prior treatment, which restricts the number of eligible cases and necessitates close collaboration with clinical partners and biobank facilities.

Another significant limitation concerns the variability in tumor engraftment efficiency. Breast tumors with high stromal or fibrotic content tend to exhibit poor take rates in NSG mice, introducing a bias toward more cellular or aggressive tumor types that are more likely to grow in vivo. Even when engraftment is successful, not all samples produce stable and expandable PDX lines, as some tumors may initially grow but fail to propagate across passages or after cryopreservation.^2^

A further challenge arises from the progressive loss of intratumoral heterogeneity that may occur during serial transplantation. Clonal selection can favor dominant cell populations, reducing the genetic and phenotypic diversity present in the original tumor and thereby limiting the representativeness of the model. To mitigate this effect, it is advisable to minimize the number of passages, ideally not exceeding passage five, and to cryopreserve early passages for future re-derivation if needed.^4^^,^^5^

This protocol uses nab-paclitaxel as a proof-of-concept agent to induce and validate chemoresistance. Although the workflow is drug-agnostic and can be adapted to other cytotoxic therapies (e.g., platinum-based or anthracycline/cyclophosphamide regimens), dose and schedule must be re-optimized for each compound. While several studies have successfully dosed NSG mice with a range of chemotherapies, scid-based strains such as NSG mice may exhibit increased sensitivity to DNA-damaging agents compared with microtubule-targeting drugs, which can limit tolerability and influence experimental design. In instances where treatment remains persistently toxic despite dose optimization, alternative immunodeficient strains with increased resistance to genotoxic stress, such as NRG mice, may be preferred; however, this was not evaluated in the present protocol.

In addition, the immunodeficient NSG strain used for these xenografts is highly susceptible to opportunistic infections, which can affect animal welfare and experimental outcomes. Strict aseptic technique and prophylactic antibiotic use are therefore essential to minimize losses. Finally, the entire process is resource-intensive and time-consuming, requiring specialized infrastructure, trained personnel, and long-term financial support. Despite these limitations, chemoresistant PDX models remain an invaluable tool for studying tumor biology and for testing novel therapeutic strategies under clinically relevant conditions.

## Troubleshooting

### Problem 1

Mice show poor condition or skin ulceration (related to Step 5).

NSG mice are highly susceptible to opportunistic infections and postoperative complications. In addition, rapid tumor growth may lead to skin ulceration, increasing the risk of local or systemic infection and compromising animal welfare.

### Potential solution

• Euthanize affected animals to prevent infection spread within the colony, particularly when ulceration is present.
• Transfer the tumor fragment to a healthy recipient mouse and monitor closely.
• Test excised tumor tissue for microbial contamination.
• Perform all surgical procedures under sterile conditions and administer prophylactic amoxicillin postoperatively, as described in the protocol.

### Problem 2

Low tumor engraftment efficiency after implantation (related to Step 8).

Tumors with high stromal or fibrotic content may exhibit reduced take rates in NSG mice, limiting successful PDX establishment.

### Potential solution

• Implant tumor fragments with high cellular content and avoid necrotic areas.
• Ensure minimal handling and avoid compressing the tumor fragment during implantation.
• Consider increasing the number of recipient mice per passage when tissue availability permits.

### Problem 3

Clonal selection during serial passaging reduces intratumoral heterogeneity (related to Note in Step 9).

Successive passages can favor expansion of dominant subclones, reducing the genetic and phenotypic diversity of the original tumor.

### Potential solution

• Limit the number of passages to a maximum of P5.
• Cryopreserve tumor fragments from early passages (e.g., P2–P3).
• Perform routine histopathological comparisons between PDXs and the original tumor to detect phenotypic drift.

### Problem 4

Loss of chemoresistance during serial passaging (related to 20d).

In the absence of sustained selective pressure, drug-sensitive subclones may expand, leading to partial or complete loss of resistance.

### Potential solution

• Perform functional drug-response assays at each passage.
• Maintain a biobank of validated chemoresistant PDX samples.
• Re-derive the model from early cryopreserved passages if resistance is lost.

### Problem 5

High toxicity or poor tolerability during drug treatment (related to Step 21).

Some chemotherapeutic agents may cause excessive toxicity in NSG mice, affecting animal health and confounding treatment-response evaluation.

### Potential solution

• Re-optimize drug dose and schedule to improve tolerability.
• Monitor body weight and clinical signs closely during treatment.
• Consider alternative dosing regimens or drug formulations validated for *in vivo* use.

## Resource availability

### Lead contact

Further information and general protocol details are available from the lead contact, Eva M. Galán-Moya (evamaria.galan@uclm.es). The lead contact can provide clarifications on the study design, experimental workflow, and overall methodological rationale.

### Technical contact

Requests for reagents, material resources, and technical questions related to protocol execution should be directed to the technical contact, David Tébar-García (david.tebar@uclm.es), who can provide detailed guidance on reagent use, experimental conditions, and troubleshooting.

### Materials availability

This study did not generate new unique reagents. All materials used in this protocol are commercially available and listed in the key resources table. Additional information required to reanalyze the data reported in this protocol is available from the lead contact upon reasonable request.

PDX models generated using this protocol are available from the lead contact upon reasonable request, subject to institutional approval, ethical regulations, and material transfer agreements.

### Data and code availability

This paper does not report original code.

## Acknowledgments

This work was supported by MCIN, ISCIII and co-funded by the European Union Project (PI22/01793), by MICIU/AEI/10.13039/501100011033, and by the European Union NextGenerationEU/PRTR (CNS2022-136040). D.T.-G. was funded by Fundación Asociación Española Contra el Cáncer (LABAE211626RODI) and Junta de Comunidades de Castilla-La Mancha (SBPLY/23/180225/000162). R.L.-R. and P.S.-O. were funded by MICIU/AEI/10.13039/501100011033 and by the European Union NextGenerationEU/PRTR (CNS2022-136040). A.P.-S. was funded by AECC Foundation (PRDAB234297PINE). P.C.-C. was funded by Diputación de Albacete (PP_RD 63/2006) and “STOP Cancer Project.”

We thank the Surgery and Anatomical Pathology services, as well as the Biobank of the General University Hospital of Albacete, for their collaboration in patient selection and sample collection.

The graphical abstract was created using BioRender.com, as indicated in the key resources table.

## Author contributions

D.T.-G., investigation, methodology, visualization, and writing – original draft; R.B.-A., E.S.-L., and M.G.-J., investigation and resources; M.G.P.-M., A.D.-P., A.P.-S., and R.L.-R., investigation and methodology; P.S.-O. and P.C.-C., investigation and visualization; V.R., supervision and writing – review and editing; E.M.G.-M.., conceptualization, funding acquisition, project administration, supervision, and writing – review and editing.

## Declaration of interests

The authors declare no competing interests.

## Declaration of generative AI and AI-assisted technologies in the writing process

During the preparation of this work, the authors used ChatGPT (OpenAI, GPT-5 model) to assist in language editing, improving clarity, and ensuring consistency of technical terminology. After using this tool, the authors carefully reviewed and edited all generated content and take full responsibility for the content of the final manuscript.
